# Synergistic defecation effects of *Bifidobacterium animalis* subsp. *lactis* BL-99 and fructooligosaccharide by modulating gut microbiota

**DOI:** 10.3389/fimmu.2024.1520296

**Published:** 2025-01-09

**Authors:** Qi Zhang, Wen Zhao, Jie Luo, Shaoqi Shi, Xiaokang Niu, Jian He, Yicheng Wang, Zhaozhong Zeng, Qiuyue Jiang, Bing Fang, Juan Chen, Yixuan Li, Fuqing Wang, Jingjing He, Jie Guo, Ming Zhang, Liwei Zhang, Shaoyang Ge, Wei-Lian Hung, Ran Wang

**Affiliations:** ^1^ Key Laboratory of Functional Dairy, Co-Constructed by Ministry of Education and Beijing Municipality, Department of Nutrition and Health, China Agricultural University, Beijing, China; ^2^ College of Food Science and Technology, Hunan Agricultural University, Changsha, China; ^3^ Probiotics R&D Department, Inner Mongolia National Center of Technology Innovation for Dairy Co. Ltd., Hohhot, China; ^4^ Yili Innovation Center, Inner Mongolia Yili Industrial Group Co. Ltd., Hohhot, China; ^5^ Department of Food Science, Tibet Tianhong Science and Technology Co., Ltd., Lhasa, China; ^6^ School of Food and Chemical Engineering, Beijing Technology and Business University, Beijing, China; ^7^ Probiotics R&D Department, Hebei Engineering Research Center of Animal Product, Sanhe, China

**Keywords:** *Bifidobacterium animalis* subsp. *lactis* BL-99, fructooligosaccharide, gut microbiota, constipation, serum inflammatory factors

## Abstract

**Introduction:**

Synbiotics have revealed the possibility of improving constipation through gut microbiota. The synergistic efficacy of *Bifidobacterium animalis* subsp. lactis BL-99 (BL-99) and fructooligosaccharide (FOS) on constipation have not been investigated.

**Methods:**

Loperamide-induced constipated mice model was established to explore the effect of BL-99, FOS, and BL-99+FOS on changes of defecation-related parameters, gut microbiota and metabolites.

**Results and discussion:**

The results showed that BL-99, FOS, and BL-99+FOS each alleviated constipation, with the synbiotic showing significant efficacy in the first black stool defecation time, fecal number, fecal weight, and the gastrointestinal transit rate (*P* < 0.05). Additionally, significant increased in serum 5-HT and IL-10 were observed in the BL-99+FOS group, alongside an increased relative abundance of *Lachnospiraceae_NK4A136_group, Blautia*, and *Clostridium sensu stricto 1*, while significantly reducing the relative abundance of *Alistipes* and *Bacteroides*. These changes facilitated alterations in short-chain fatty acids (SCFAs) metabolism, and were closely associated with the expression of genes related to the 5-HT pathway and the modulation of serum inflammatory factors. This study provides a theoretical basis for BL-99 and FOS synbiotics to improve constipation by regulating the gut microbiota and metabolites.

## 1 Introduction

Constipation, a prevalent gastrointestinal disorder characterized by infrequent bowel movements, hardened stools, and discomfort during defecation, affects approximately 10.1% of the global population according to a study based on the Rome IV criteria, with its incidence on the rise, particularly due to demographic aging trends (1, 2). This multifaceted syndrome not only compromises physiological well-being, but also imposes substantial psychological and societal burdens. Chronic constipation can lead to severe gastrointestinal complications, including colorectal cancer, heightened cardiovascular risks, and increased susceptibility to diabetes, significantly impacting individuals’ quality of life and escalating healthcare costs (3, 4). The pathophysiology of constipation is intricate, involving various mechanisms within the gastrointestinal tract. Central among these is impaired intestinal smooth muscle motility, which plays a pivotal role in facilitating bowel movements (5). Disruptions in the enteric nervous system (ENS), alongside alterations in neurotransmitter levels and intestinal dysbiosis, contribute to delayed colonic transit times and, consequently, constipation symptoms (6–8).

Given the growing recognition of the gut microbiota’s role in health and disease, therapeutic interventions have expanded to encompass microbiota-targeted approaches such as synbiotics (9). These formulations, combining probiotics and prebiotics, aim to restore microbial equilibrium, foster the proliferation of beneficial bacteria, and suppress pathogenic strains, thereby ameliorating bowel function (10, 11). The manipulation of gut microbiota has emerged as a promising avenue for managing constipation, with synbiotics showing particular efficacy in modulating the gut milieu and alleviating associated symptoms (12). Synbiotics harness the synergistic interactions between prebiotics and probiotics to modulate the gut microenvironment, promote microbial homeostasis, and mitigate constipation (13, 14). Prebiotics, selectively fermentable substrates, stimulate the growth and activity of beneficial microbial communities, predominantly *Bifidobacterium* and *Lactobacillus*, thereby enhancing fecal bulk and water content, which softens stools and facilitates transit (13, 14). Concurrently, probiotics introduce live beneficial microorganisms that bolster gut microbiota diversity, reinforce intestinal barrier integrity, and regulate immune responses (15). The combined administration of prebiotics and probiotics in synbiotic formulations augments these effects, particularly in fostering the production of short-chain fatty acids (SCFAs), essential for mucosal health and intestinal motility regulation (9, 16). Empirical evidence from clinical and experimental studies corroborates the efficacy of synbiotics in constipation management, with specific formulations, including fructooligosaccharides (FOS) and galactooligosaccharides (GOS) paired with *Bifidobacterium* strains, demonstrating significant reductions in gut transit time, increased stool frequency, and improved consistency (4). Moreover, synbiotic interventions have shown favorable effects on the gut-brain axis, potentially alleviating abdominal discomfort associated with constipation (9, 17).

*Bifidobacterium animalis* subsp. *lactis* BL-99 (BL-99), a probiotic strain isolated from the infant’s gut, has demonstrated substantial promise in regulating gut health (18, 19). It achieves this through modulating the gut microbiota, influencing neurotransmitter levels, reducing inflammation, and interacting beneficially with prebiotics such as FOS. In an *in vitro* fermentation model, the combination of BL-99 and FOS has synergistic effects, enhancing the probiotic’s ability to modulate gut microbiota and produce beneficial metabolites. The study has demonstrated the synergistic effects of FOS and BL-99, promoting a significant increase in acetic acid content and a marked decrease in CO_2_ and H_2_S contents in the fermentation broth. Furthermore, the BL-99 and FOS combination significantly altered the structure of the gut microbiota, enhancing the relative abundances of beneficial bacteria such as *Bifidobacterium*, *Fecalibacterium*, *Lactobacillus*, *Subdoligranulum*, and *Blautia*, and decreasing those of harmful bacteria including *Bilophila* and *Escherichia-Shigella (* 20).​ These findings suggested that BL-99 and FOS synergistically regulated the composition and structure of the gut microbiota, increasing acetic acid and decreasing CO_2_ and H_2_S levels, thereby providing a theoretical basis for the application of synbiotics.

This study aims to systematically evaluate the synergistic efficacy of BL-99+FOS in a constipated mice model, investigating their impact on fecal parameters, colonic morphology, serum inflammatory markers, and neurotransmitter levels. Through a comprehensive analysis of gut microbiota composition and short-chain fatty acid metabolic pathways, and related gene expressions, this research seeks to elucidate the mechanisms by which FOS and BL-99 synergistically ameliorate constipation. By employing a multifaceted methodological approach, this investigation endeavors to provide critical insights into the potential of synbiotic interventions to modulate gut health, thereby contributing to the development of innovative and effective therapeutic strategies for constipation management.

## 2 Materials and methods

### 2.1 Animals and experimental design

Thirty-six 6-week-old specific pathogen-free (SPF) female BALB/c mice were purchased from Vital River Laboratory Animal Technology Co., Ltd (Being, China). The mice were acclimatized for one week under SPF conditions at the China Agricultural University’s facility, with a 12 h light-dark cycle, at 25 ± 2°C and 50% ± 5% humidity. The experimental design was approved by the China Agricultural University’s Ethical Committee (Approval No. Aw61103202-5-1), in accordance with the EU Directive 2010/63/EU.

### 2.2 Bacterial strain

BL-99 was sourced from the China General Microbiological Culture Collection Center (CGMCC 15650).

### 2.3 Experimental procedure

Mice were randomly divided into six groups (n = 6/group): control (Ctrl), constipation model (LOP), positive control (POS), BL-99, FOS, and a synbiotic combination of BL-99 and FOS (BL-99+FOS). Except for the Ctrl group, all groups received intragastric administration of loperamide hydrochloride (10 mg/kg) from days 8 to 17 following a 7-day acclimatization period to induce constipation. Upon successful modeling, treatments were administered from days 18 to 31. The control and model groups received saline, the FOS group received FOS solution (700 mg/kg), the BL-99 group received BL-99 bacterial suspension (5×10^9^ CFU/day), and the synbiotic group received a mixture of BL-99 and FOS (700 mg/kg FOS + 5×10^9^ CFU/day BL-99). The positive drug control group received polyethylene glycol 4000 solution (0.17 g/kg).

### 2.4 Fecal parameters measurement

With reference to the research of Lim YJ et al., the fecal parameters were carried out, which is briefly described as follows (21). After fasting for 16 h, mice were gavage with 0.2 mL activated carbon powder solution to record the first black stool discharge time, and the number of mice defecation granules within 2 h. Fresh feces were dried at 105 °C for 16 h, and the fecal moisture content is calculated according to the following formula: Fecal moisture content = (W_wet_ - W_dry_)/W_wet_ × 100%.

### 2.5 Gut motility experiment

After fasting for 16 h, 0.1 mL carmine solution was administered orally. Mice were euthanized 30 min later, and the entire intestine was dissected. The lengths of the intestine (S_1_) and the distance traveled by the carmine marker (S_2_) were measured to calculate the propulsion rate with the formula D = S_2_/S_1_×100%.

### 2.6 Histological analysis

Tissues were dehydrated, embedded, and sectioned for hematoxylin and eosin (H&E) staining to assess colon histological changes.

### 2.7 Serum indexes analysis

Serum levels of neurotransmitters and cytokines, including serotonin (5-HT), acetylcholine (ACh), substance P (SP), vasoactive intestinal peptide (VIP), nitric oxide synthase (NOS), Interleukin-10 (IL-10), Interleukin-4 (IL-4), Interleukin-6 (IL-6), and tumor necrosis factor-α (TNF-α) were quantified using ELISA (Shanghai Enzyme-linked Biotechnology Co., Ltd., China), following the manufacturer’s instructions.

### 2.8 Microbiota analysis of mouse cecal contents

#### 2.8.1 DNA extraction and PCR amplification

Microbial DNA was extracted from mouse cecal contents using the HiPure Soil DNA Kit (Magen, Guangzhou, China) following the manufacturer’s instructions. The V3-V4 regions of the 16S rRNA gene were amplified using primers 341F and 806R with the following PCR conditions: initial denaturation at 95°C for 3 min, followed by 30 cycles of denaturation at 95°C for 30 s, annealing at 55°C for 30 s, and extension at 72°C for 30 s, with a final extension at 72°C for 5 min.

#### 2.8.2 Illumina sequencing

PCR products were purified, quantified, and pooled in equimolar amounts. The library was prepared according to the Illumina protocol and sequenced on an Illumina MiSeq platform for 250 bp paired-end reads.

### 2.9 SCFAs quantification

#### 2.9.1 Standard preparation

A mixed standard solution containing acetic acid, propionic acid, and butyric acid (Sigma-Aldrich, Germany, Chromatographic grade) was prepared in butanol. Internal standard (2-ethylbutyric acid) was added to each sample before analysis.

#### 2.9.2 Gas Chromatography-Mass Spectrometry (GC-MS)

Samples were analyzed using a GC-MS system equipped with a flame ionization detector. DB-FFAP column (0.32 mm×30 mm×0.5 μm) was used and the temperature was programmed from 80°C to 220°C. SCFAs were identified based on their retention times and quantified using calibration curves.

### 2.10 Metabolomic analysis

#### 2.10.1 Sample preparation

Cecal contents were homogenized in methanol: water (4:1) containing internal standards. Samples were centrifuged, and the supernatant was analyzed.

#### 2.10.2 LC-MS/MS analysis

Chromatographic separation was performed on an ACQUITY UPLC HSS T3 column using a gradient elution of water and acetonitrile, both with 0.1% formic acid. Mass spectrometry was conducted in both positive and negative ion modes on a Q Exactive HF-X mass spectrometer (18).

### 2.11 qPCR analysis of colonic gene expression

#### 2.11.1 RNA extraction and cDNA synthesis

Total RNA was extracted from colonic tissues using Trizol reagent. cDNA was synthesized from 1 µg of total RNA using the PrimeScript RT Reagent Kit (Takara, Japan).

#### 2.11.2 Real-time PCR Analysis

qPCR was performed using SYBR Green Master Mix on a LightCycler 480 System. Primers for target genes (*GAPDH, Tph1, TGR-5, Fxr, Vdr*) were designed based on mouse sequences. The relative expression levels of target genes were normalized to GAPDH and calculated using the 2^(-ΔΔCt) method (22).

### 2.12 Statistical analysis

Data were expressed as mean (M) ± standard deviation (SD). Differences between groups were analyzed using one-way ANOVA followed by LSD *post-hoc* test for multiple comparisons. A *P*-value < 0.05 was considered statistically significant. Statistical analyses were performed using GraphPad Prism version 9.5.0.

## 3 Results

### 3.1 FOS or BL-99 alone or combined alleviate defecation-related parameters in constipated mice

**Figure 1:**
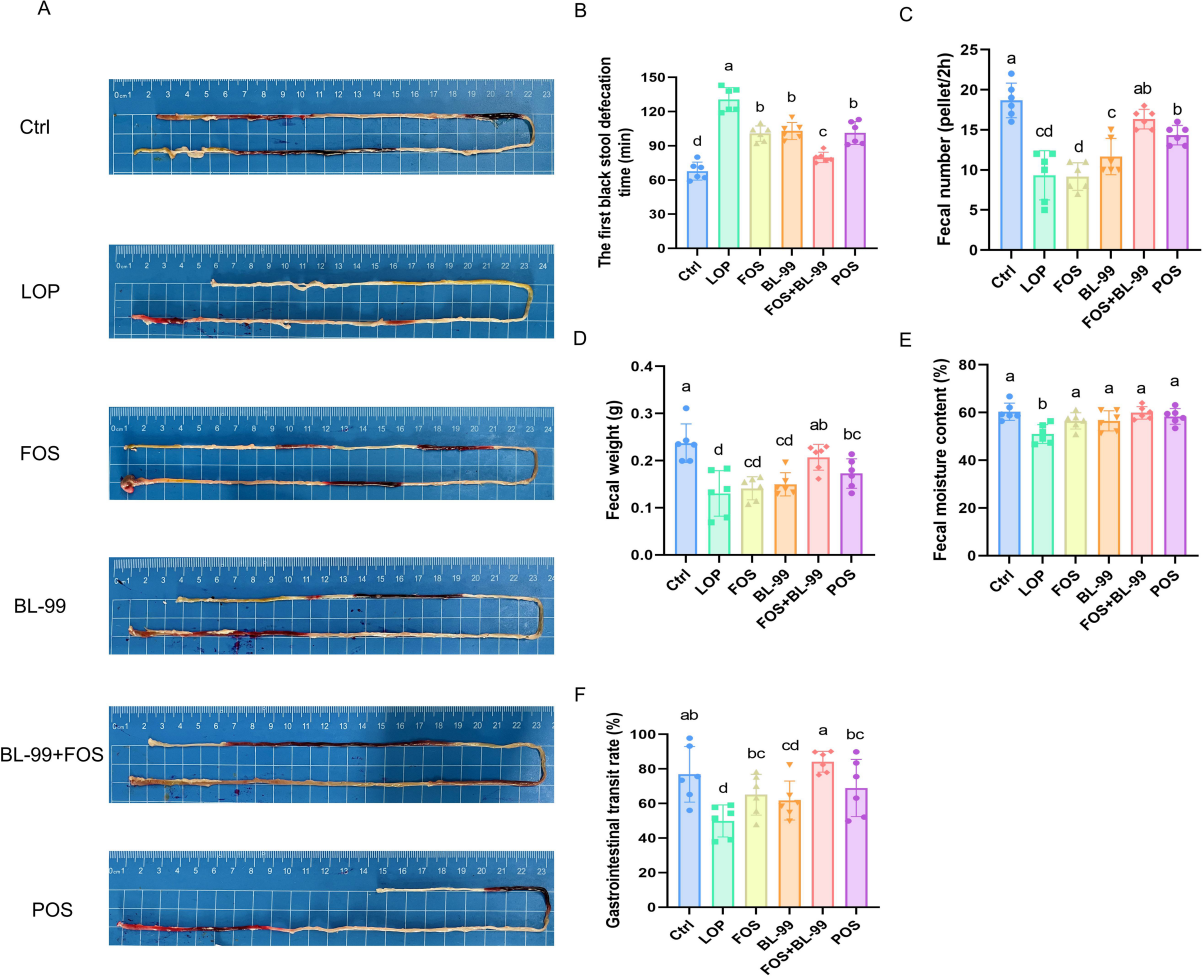
FOS or BL-99 alone or combined alleviate defecation-related parameters in constipated mice. **(A, B)** The first black stool defecation time, **(C)** fecal number, **(D)** fecal weight, **(E)** fecal moisture content, and **(F)** gastrointestinal transit rate. Values were expressed as M ± SD (n = 6). Results were compared by one-way ANOVA followed by LSD *post-hoc* test. Different letters represent significant differences (*P* < 0.05).

To evaluate the efficacy of prebiotics, probiotics, and synbiotics, mice were pretreated with loperamide for two weeks, followed by administration of FOS, BL-99, and BL-99+FOS for another two weeks. Loperamide treatment significantly induced constipation, evidenced by prolonged first black stool defecation time, reduced fecal number, lower fecal weight, decreased fecal moisture content, and decreased gastrointestinal transit rate compared to the Ctrl group (*P* < 0.05, **Figure 1**). Compared with the LOP group, the FOS, BL-99, FOS+BL-99, and POS groups all alleviated defecation-related parameters. Compared to the FOS and BL-99 groups, the FOS+BL-99 group significantly decreased the first black stool defecation time, and significantly increased the fecal number, fecal weight, and the gastrointestinal transit rate (*P* < 0.05). Although the BL-99+FOS group did not show significant differences in some defection indexes such as fecal number, fecal weight, and fecal moisture content, there was still a trend of improvement compared with the FOS and BL-99 groups.

**Figure 2:**
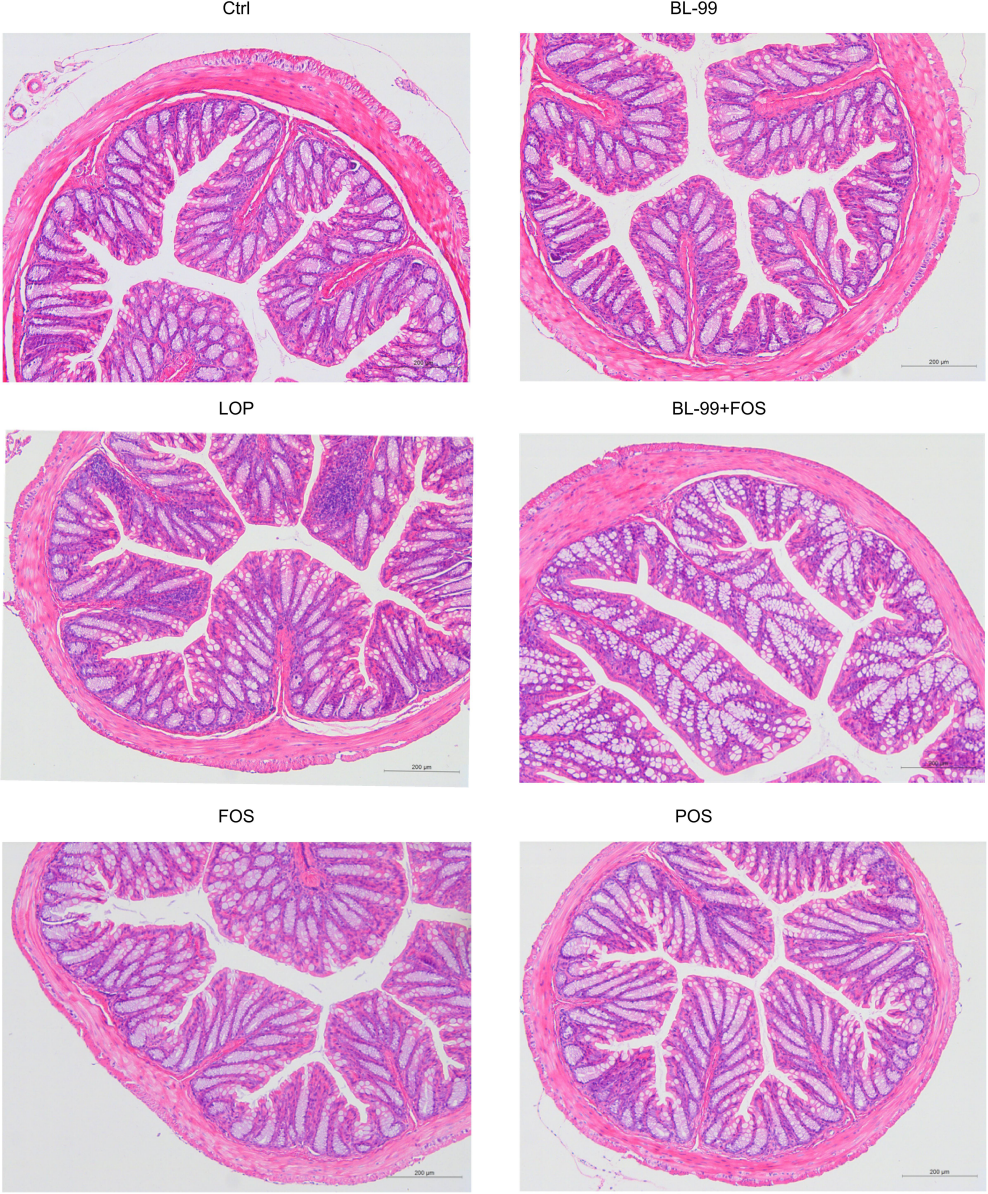
Morphology of the colon stained with hematoxylin and eosin (H&E).

Histological staining was employed to assess alterations in colonic histomorphology (**Figure 2**). An intact colonic structure was observed in the normal control group, while the constipation model exhibited reduced goblet cells and inflammatory infiltrations. Compared to the constipation model group, colonic sections from the FOS group, BL-99 group, and FOS+BL-99 group showed a restoration of villus structure, resembling morphology observed in the Ctrl group.

### 3.2 FOS or BL-99 alone or combined changed neurotransmitters and inflammatory cytokines levels in the serum of constipated mice

**Figure 3:**
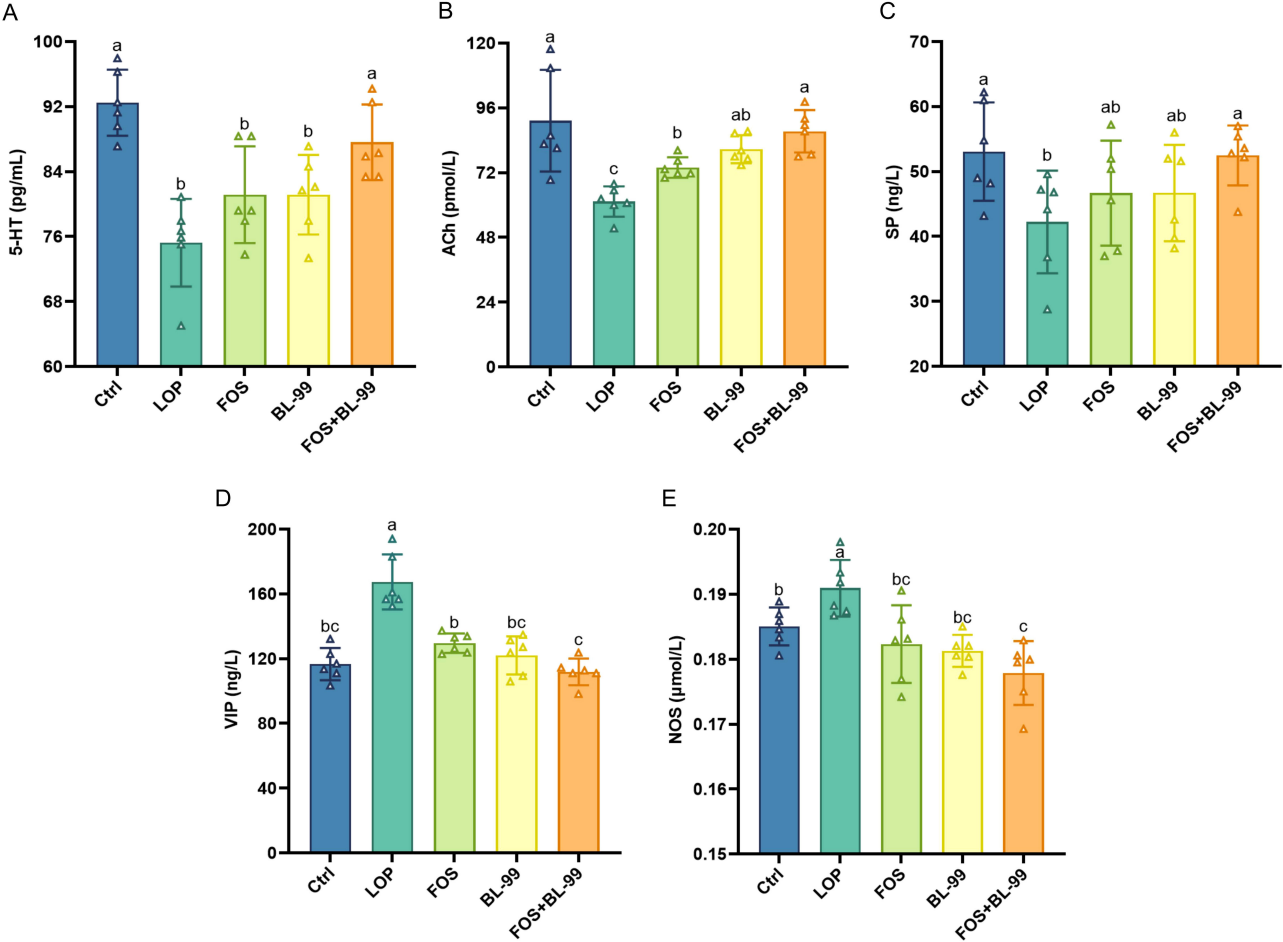
The effects of BL-99, FOS, BL-99+FOS on serum neurotransmitter levels in constipated mice. **(A)** Serotonin (5-HT), **(B)** Acetylcholine (ACh), **(C)** Substance P (SP), **(D)** Vasoactive Intestinal peptide (VIP), **(E)** Nitric oxide synthase (NOS). Values were expressed as M ± SD (n = 6). Results were compared by one-way ANOVA followed by LSD *post hoc* test. Different letters represent significant differences (*P* < 0.05).

Through the measurement of the excitatory neurotransmitters 5-HT, ACh, and SP, and the inhibitory neurotransmitters VIP and NOS, we explored whether treatment with FOS or BL-99 alone or combined can ameliorate intestinal function by modulating the levels of intestinal neurotransmitters (**Figure 3**). In terms of excitatory neurotransmitters, the serum levels of 5-HT, SP, and ACh were significantly lower in the LOP group mice compared to the Ctrl group (*P* < 0.05). Treatment with FOS or BL-99 alone significantly increased the levels of ACh (*P* < 0.01) and showed a trend towards modulating 5-HT and SP levels, although these changes were not statistically significant compared to the LOP group. Notably, the combined use of BL-99 and FOS significantly increased the levels of 5-HT and ACh (*P* < 0.05) compared to the LOP group, more effectively restoring the balance of intestinal neurotransmitters and potentially promoting the recovery of intestinal function. In terms of inhibitory neurotransmitters, the serum levels of VIP and NO were significantly elevated in the LOP group mice (*P* < 0.05). Treatment with BL-99, FOS, and their combination all effectively reduced the levels of VIP and NO (*P* < 0.05), with the combined treatment showing the most significant reduction (*P* < 0.01). These findings indicate that both individual and combined treatments of FOS and BL-99 can modulate intestinal neurotransmitter levels, with the combined treatment showing the most pronounced effects in restoring excitatory neurotransmitter levels and reducing inhibitory neurotransmitter levels, thereby potentially ameliorating intestinal function.

**Figure 4:**
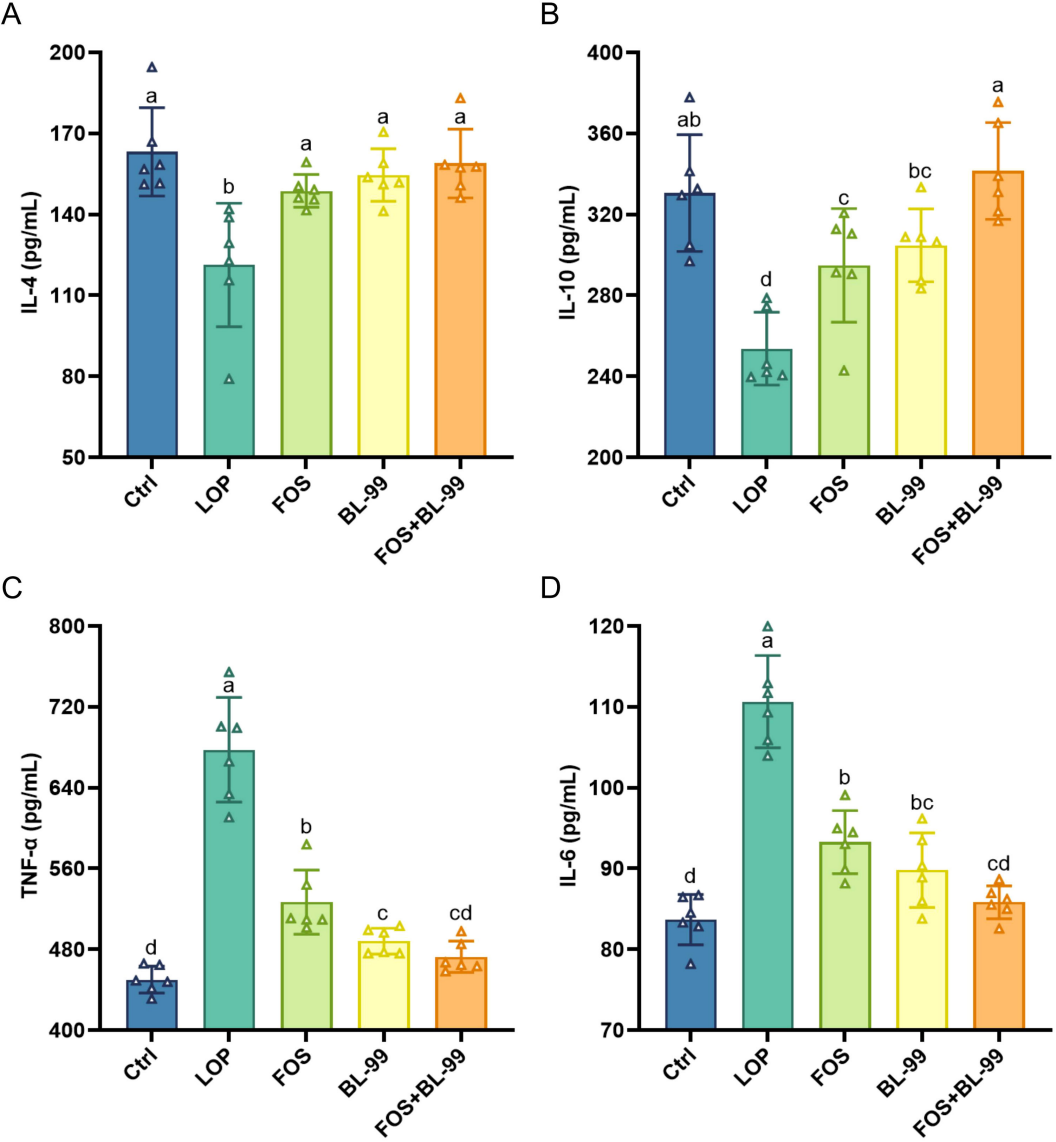
The effects of BL-99, FOS, BL-99+FOS on inflammatory cytokines levels in constipated mice. **(A)** IL-4, **(B)** IL-10, **(C)** Tumor necrosis factor-α (TNF-α), **(D)** IL-6. Values were expressed as M ± SD (n = 6). Results were compared by one-way ANOVA followed by LSD *post hoc* test. Different letters represent significant differences (*P* < 0.05).

The serum levels of the anti-inflammatory cytokines IL-4 and IL-10, as well as the pro-inflammatory cytokines TNF-α and IL-6, were assessed to quantify the effects of prebiotics, probiotics, and synbiotics on inflammatory factors in constipated mice (**Figure 4**). In constipated mice induced by loperamide hydrochloride, there was a statistically significant decrease in the levels of the anti-inflammatory cytokines IL-4 and IL-10 in the constipated mice compared to the control group. This indicates an imbalance in inflammatory cytokines, with a dominance of pro-inflammatory cytokines in the serum of constipated mice. The administration of FOS or BL-99 independently, or in combination, significantly increased the serum levels of the anti-inflammatory cytokines IL-4 and IL-10 and reduced the pro-inflammatory cytokine TNF-α and IL-6 (*P* < 0.05). The combination of BL-99 and FOS showed a synergistic effect, with a significantly greater increase in IL-10 levels compared to BL-99 or FOS used alone. This suggests that the combination of BL-99 and FOS can effectively improve inflammation of constipated mice.

### 3.3 FOS or BL-99 alone or combined changed gut microbiota in constipated mice

**Figure 5:**
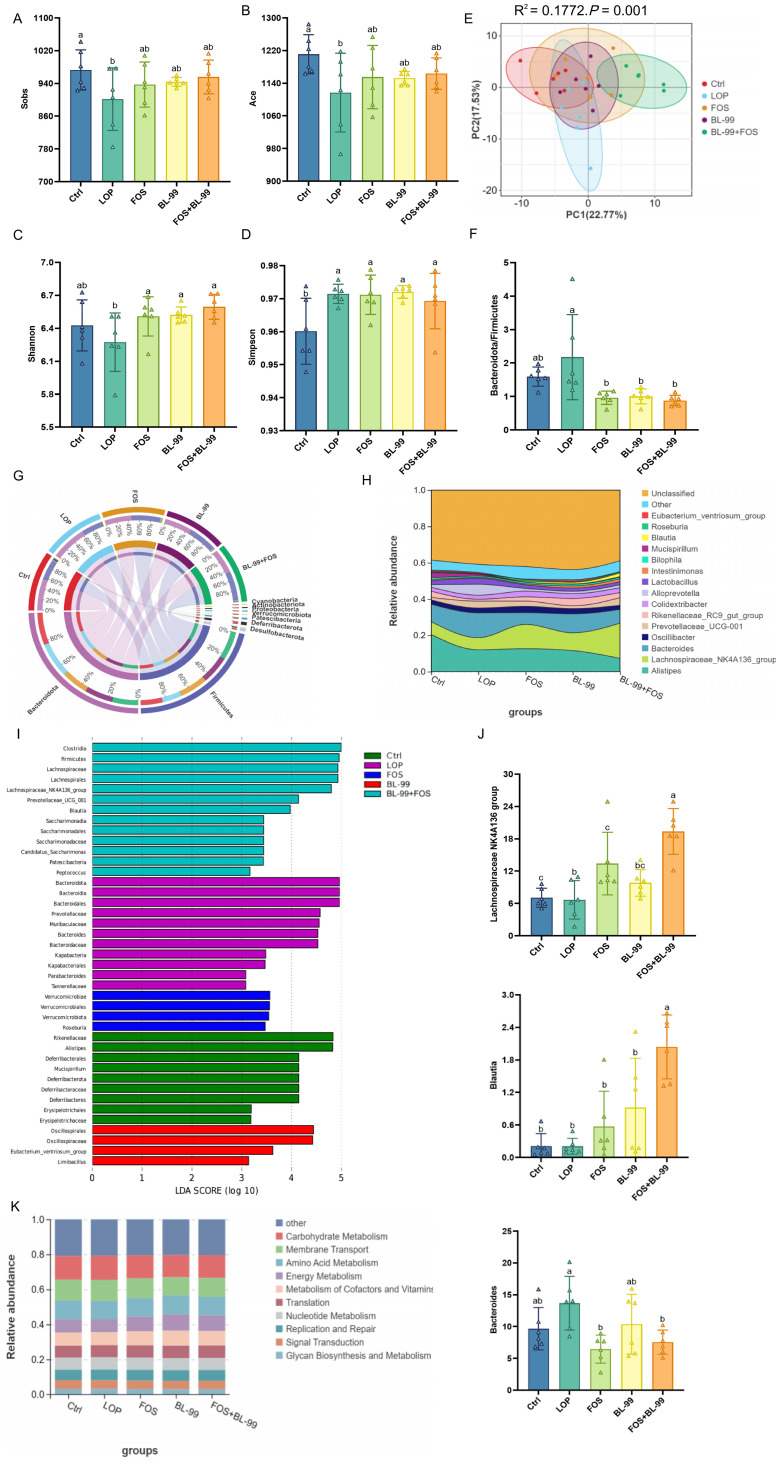
The effects of BL-99, FOS, BL-99+FOS on cecal microbiota composition in constipated mice. **(A)** Sobs index, **(B)** Ace index, **(C)** Shannon index, **(D)** Simpson index, **(E)** Principal coordinate analysis (PCoA) based on the OTU levels using Bray-Curtis distances, **(F)** The relative abundance ratio of *Actinobacteria* to *Firmicutes*, **(G)** The relative abundance of the top 10 at the phylum level. **(H)** The relative abundance of the top 10 at the genus level. **(I)** LEfSe (Linear discriminant analysis Effect Size) analysis among groups. **(J)** Tax4Fun functional abundance stacked chart **(K)**.

The cecal microbial diversity in mice was significantly enhanced by the combined treatment with BL-99 and FOS compared to the LOP group. This increase in microbial diversity and richness was further corroborated by notable improvements in alpha diversity indices like Sobs, Shannon, and Chao1 (**Figures 5A–D**). beta diversity was effectively depicted through Principal Coordinates Analysis (PCoA) using Bray-Curtis distances (**Figure 5E**). This analysis distinctly separated the microbial compositions between the Ctrl and LOP groups, particularly in mice treated with BL-99 and FOS. The Adonis test (*P* = 0.001) confirmed significant differences in community composition, highlighting the substantial impact of the treatments on the gut microbiome’s structure.

Further analysis of the predominant bacterial phyla in the intestinal microbiota across various intervention groups revealed the primary phyla as *Bacteroidetes*, *Firmicutes*, *Desulfobacterota*, *Deferribacterota*, and *Patescibacteria* (**Figure 5G**). Results showed an increase in *Bacteroidetes* and a decrease in *Firmicutes* within the LOP group. Additionally, the *Bacteroidetes*-to-*Firmicutes* ratio was significantly higher in the LOP group compared to the Ctrl group (**Figure 5F**). Post-intervention assessments revealed a significant reduction in this ratio following treatment with BL-99, FOS, and BL-99+FOS, indicating a potential restoration of microbial balance. At the genus level, the top ten in terms of abundance included *Alistipes*, *Lachnospiraceae NK4A136 group*, *Bacteroides*, *Oscillibacter*, *Prevotellaceae UCG-001*, *Rikenellaceae RC9 gut group*, *Colidextribacter*, *Alloprevotella, Lactobacillus*, and *Intestinimonas* (**Figure 5H**). Using the LEfSe method with an LDA score threshold greater than 3, significant microbial variations within the cecum contents were observed, with each group showing distinct microbial compositions (**Figure 5I**). In the LOP group, *Prevotellaceae UCG-001* and *Parabacteroides* were more abundant compared to the Ctrl group, while *Odoribacter*, *Rikenellaceae RC9 gut group*, *Alistipes*, *Mucispirillum*, and *Peptococcus* showed lower relative abundance. Notably, significant differences were observed for *Alistipes*, *Prevotellaceae*, *Rikenellaceae*, *Mucispirillum*, and *Peptococcus* (*P* < 0.05). In the BL-99+FOS group, the *Lachnospiraceae NK4A136 group*, *Blautia*, *Candida*, *Peptococcus*, *Lachnospiraceae UCG-006*, *Lachnoclostridium*, *Turicibacter*, and *Clostridium sensu stricto 1* were significantly enriched (*P* < 0.05), whereas *Alistipes*, *Bacteroides*, *Eubacterium ventriosum group*, and *Akkermansia* were significantly less abundant compared to the LOP group (*P* < 0.05). Moreover, compared to individual use of FOS and BL-99, their combination significantly enriched *Blautia* and *Clostridium sensu stricto 1* (*P* < 0.05, **Figure 5J**). The influence of synbiotic treatment on the cecal microbiota was further evidenced by alterations in community structure, impacting their metabolic pathways. To elucidate these effects, PICRUSt2 software was utilized for functional prediction analysis based on 16S rRNA gene sequences (**Figure 5K**). The KEGG Pathway enrichment bar charts indicated that primary metabolic pathways involved metabolism, genetic information processing, cellular processes, environmental information processing, organismal systems, and human diseases.

### 3.4 FOS or BL-99 alone or combined changed metabolites in constipated mice

**Figure 6:**
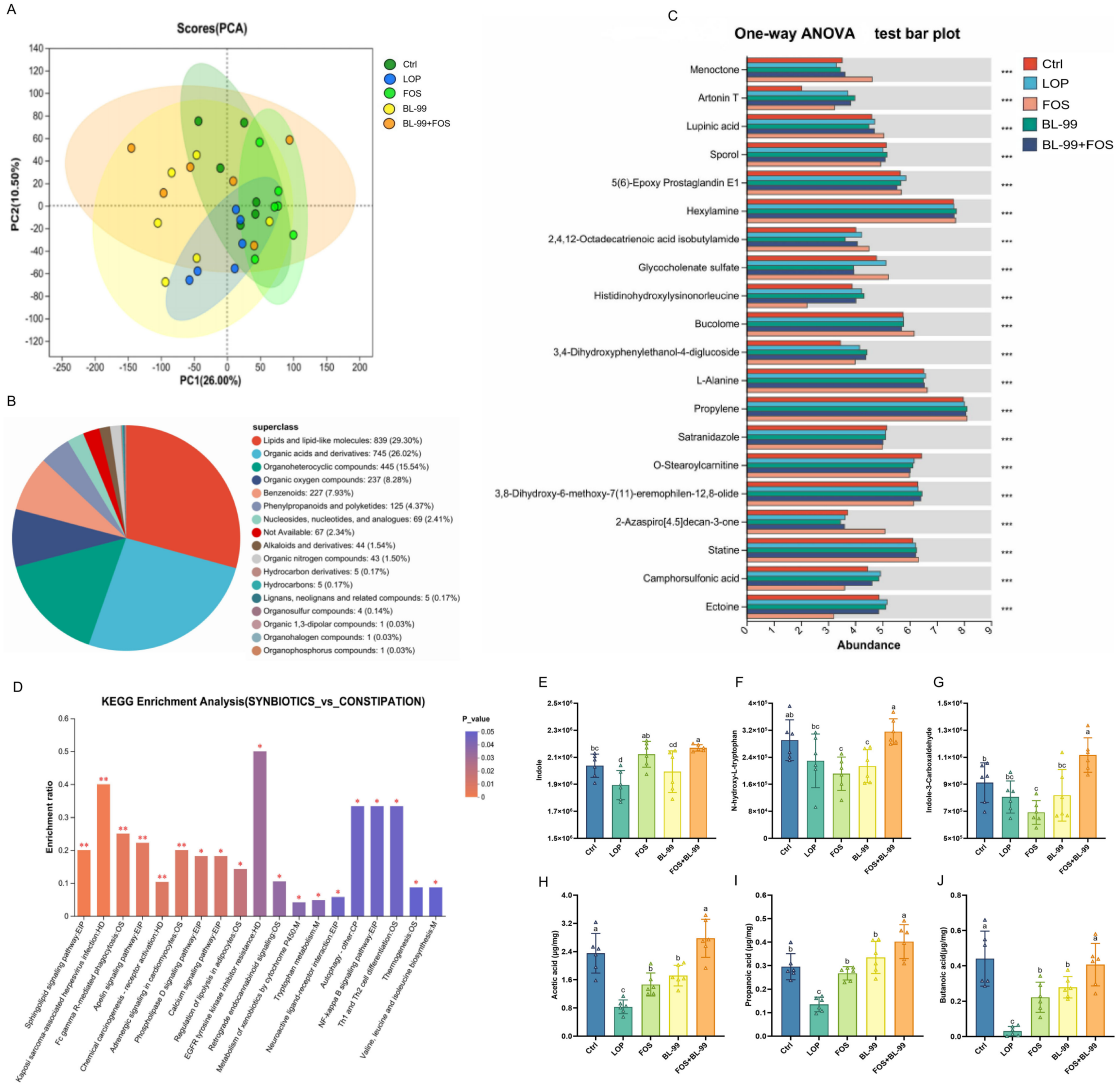
The effects of BL-99, FOS, BL-99+FOS on cecal metabolites in constipated mice. **(A)** PCA analysis of colonic metabolite samples based on LC-MS/MS data, **(B)** Hierarchical classification pie chart of HMDB compounds, **(C)** Multiple group comparison bar graphs, **(D)** KEGG enrichment analysis, **(E–G)** Differences metabolites in cecal contents among groups, **(H-J)** The discrepancy of short chain fatty acids (SCFAs) in cecal contents among groups. Data were expressed as the M ± SD (n = 6). ^*^ *P*<0.05, ^**^ *P*<0.01, ^***^ *P*<0.001. Different letters represent significant difference (P<0.05).

To explore the differences in cecal metabolites among different mouse groups, Principal Component Analysis (PCA) was initially employed to analyze the metabolic profiles of the cecal contents in mice, but PCA results not revealed good separation among groups (**Figure 6A**). From the secondary identification, all 2863 metabolites identified were indexed in the HMDB database and classified into 17 HMDB super-classes. Lipids and lipid-like molecules contained 839 metabolites, accounting for the largest category (29.30%), followed by organic acids and derivatives, which constituted 26.02% (**Figure 6B**). Our findings reveal that the combined intervention of BL-99 and FOS significantly modulated various metabolites (**Figure 6C**). The nature of these metabolic changes was significantly distinct across the different intervention approaches, with the combined treatment of BL-99 and FOS ameliorating these metabolic shifts. In the BL-99+FOS treated groups, pathways related to the metabolism of fructose and mannose, as well as the biosynthesis of phenylalanine, tyrosine, and tryptophan, tryptophan metabolism, and fatty acid biosynthesis and degradation, were significantly enhanced compared to the LOP group (*P* < 0.05, **Figure 6D**). Conversely, pathways involved in cell apoptosis and galactose metabolism were significantly reduced (*P* < 0.05).

The biosynthesis of 5-HT requires its precursor, tryptophan, which is ingested through dietary proteins and subsequently metabolized by the liver before entering the bloodstream. Once in the human body, tryptophan is initially converted into 5-HT by the enzyme tryptophan hydroxylase through an oxidative process, and then rapidly transformed into 5-HT by the enzyme 5-HT decarboxylase. Studies have identified a significant negative correlation between the levels of tryptophan and its metabolic precursors, such as 5-HT, and delayed colonic transit times. As illustrated in **Figure 6E–G**, in a constipation model using mice, the levels of tryptophan and its metabolites in the cecal contents are significantly reduced. The intervention of BL-99+FOS notably increases the levels of N-hydroxy-L-tryptophan, indole, and indole-3-aldehyde, demonstrating a statistically significant elevation (*P* < 0.05).

Dietary fibers in the intestinal tract are fermented by bacteria, predominantly producing SCFAs, which play a central role in the microbiota-gut-brain axis communication. As illustrated in **Figure 6H–J**, the levels of the three SCFAs were significantly reduced in the constipation model group compared to the control group (*P* < 0.05). All intervention groups exhibited an increase in SCFAs levels, with the combined use of BL-99 and FOS proving most effective in elevating SCFAs levels in the cecal contents of constipated mice, surpassing those of the groups using either agent alone. Overall, the joint application of BL-99 and FOS effectively promoted gastrointestinal microbial fermentation producing SCFAs.

**Figure 7:**
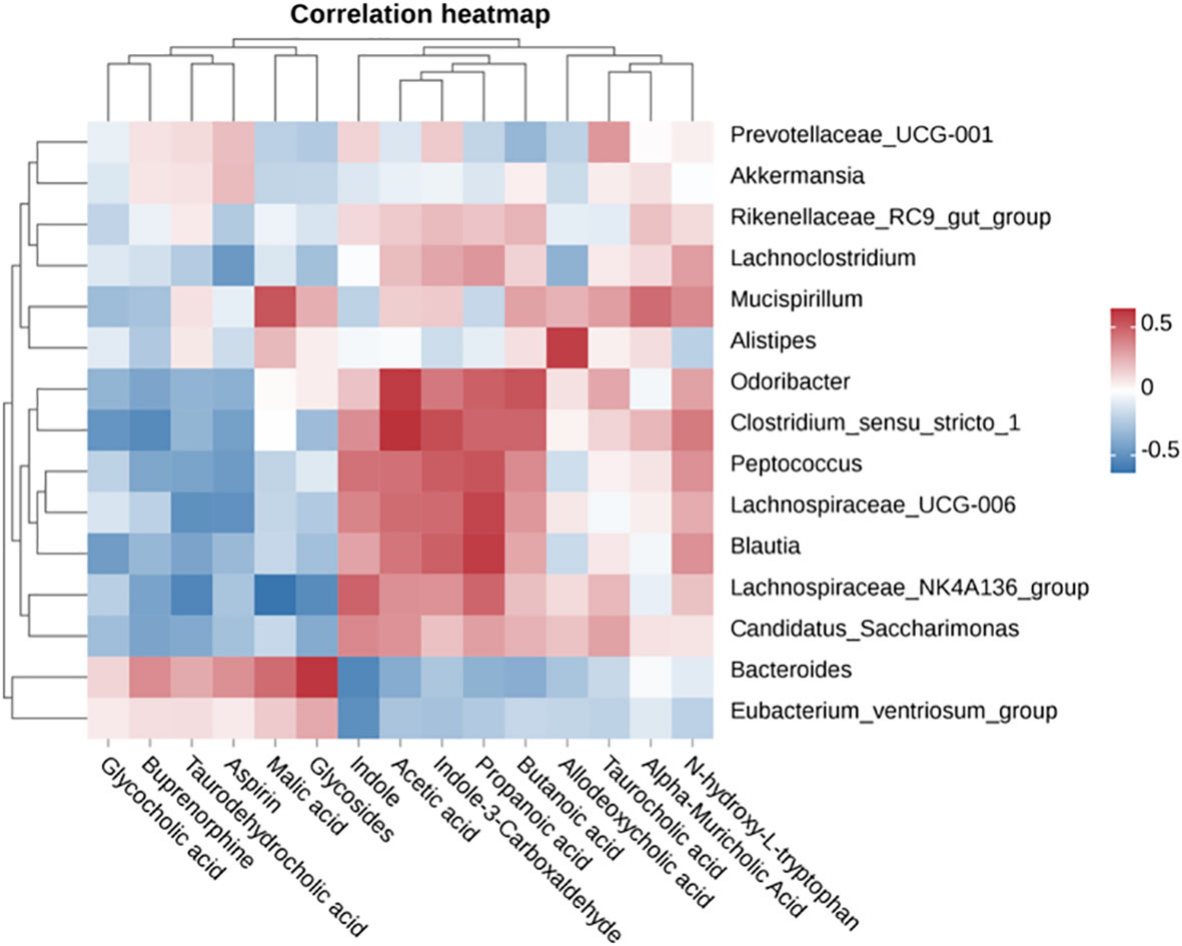
Pearson correlation heatmap analysis of the correlation between cecal metabolites and microbiota.

To investigate the interrelationships and variability among different bacterial genera and metabolites, we employed a heatmap to visually represent the clustering patterns of diverse metabolites and bacterial genera within the cecum. In **Figure 7**, we observed distinct patterns of correlation between various bacterial genera and metabolite levels: Genera such as *Bacteroides*, *Clostridium sensu stricto 1*, *Lachnospiraceae_UCG-006*, *Akkermansia*, *Lachnospira*, and *Candida* displayed a negative correlation with glycocholic acid, taurocholic acid, malic acid, glycosides, butyrate, and aspirin levels. Conversely, they exhibited a positive correlation with acetate, propionate, butyrate, N-hydroxy-L-tryptophan, deoxycholate, taurodeoxycholate, α-muricholic acid, indole, and indole-3-carboxaldehyde levels. In contrast, genera such as *Prevotella* and *Ruminococcus* demonstrated an opposite trend in relation to the aforementioned metabolite levels.

**Figure 8:**
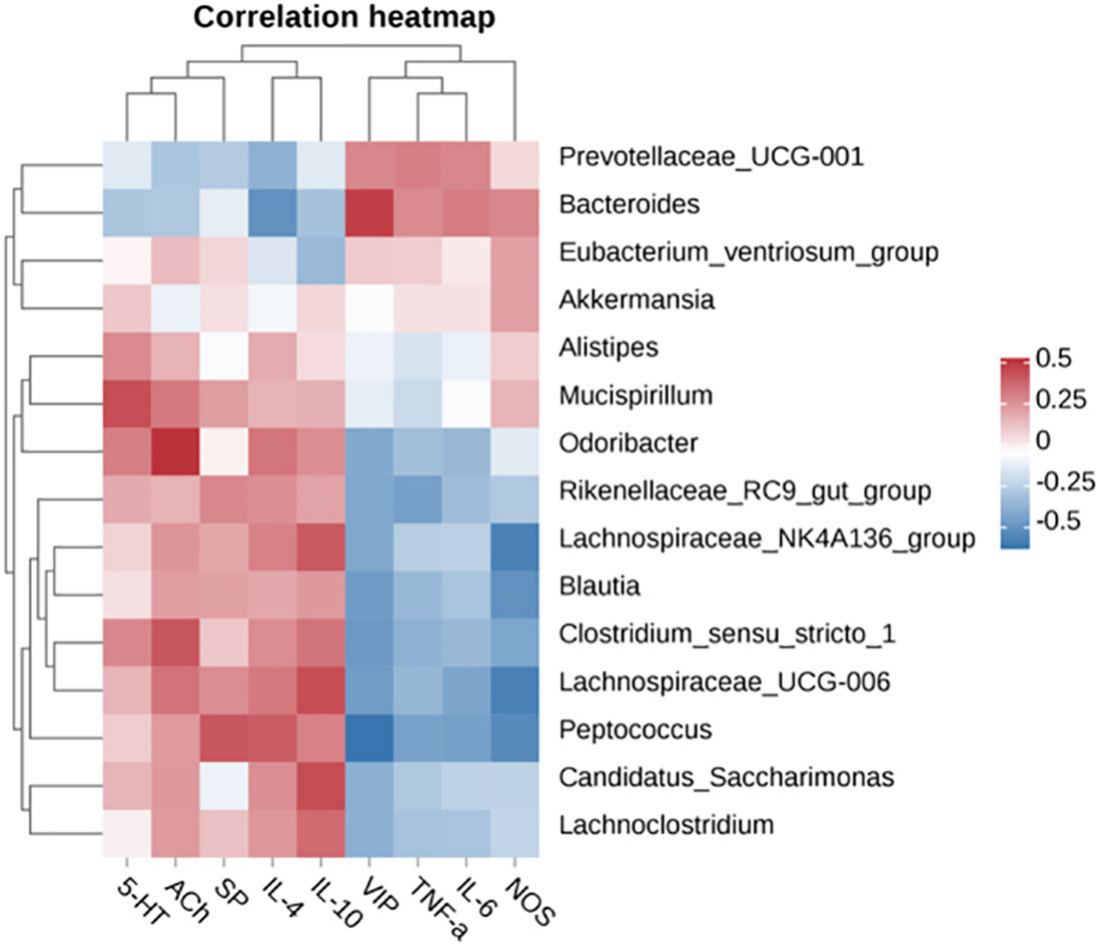
Pearson correlation heatmap analysis of the correlation between serum neurotransmitters, inflammation parameters, and cecal microbiota.

Pearson correlation analysis was further conducted to elucidate the relationship between differentially enriched microorganisms and serum neurotransmitters or inflammation parameters. *Bacteroides* enriched in the LOP group showed a strong inverse correlation with cecal IL-4 levels (*P* < 0.05) (**Figure 8**). Notably, oral administration of FOS+BL-99 resulted in significant alterations in the intestinal microbiota of mice with constipation, suggesting a pivotal role of the gut microbiota in mitigating loperamide-induced constipation. *Lachnospiraceae*_UCG-006, enriched in the FOS+BL-99 group, exhibited a significant positive correlation with serum IL-10 levels (*P* < 0.05) and a significant negative correlation with VIP, NOS, and IL-6 levels in the cecum (*P* < 0.05). These findings underscore the critical influence of FOS+BL-99 on gut microbiota modulation, highlighting its potential to alleviate constipation symptoms induced by loperamide.

### 3.5 FOS or BL-99 alone or combined altered gene transcription levels in constipated mice

**Figure 9:**
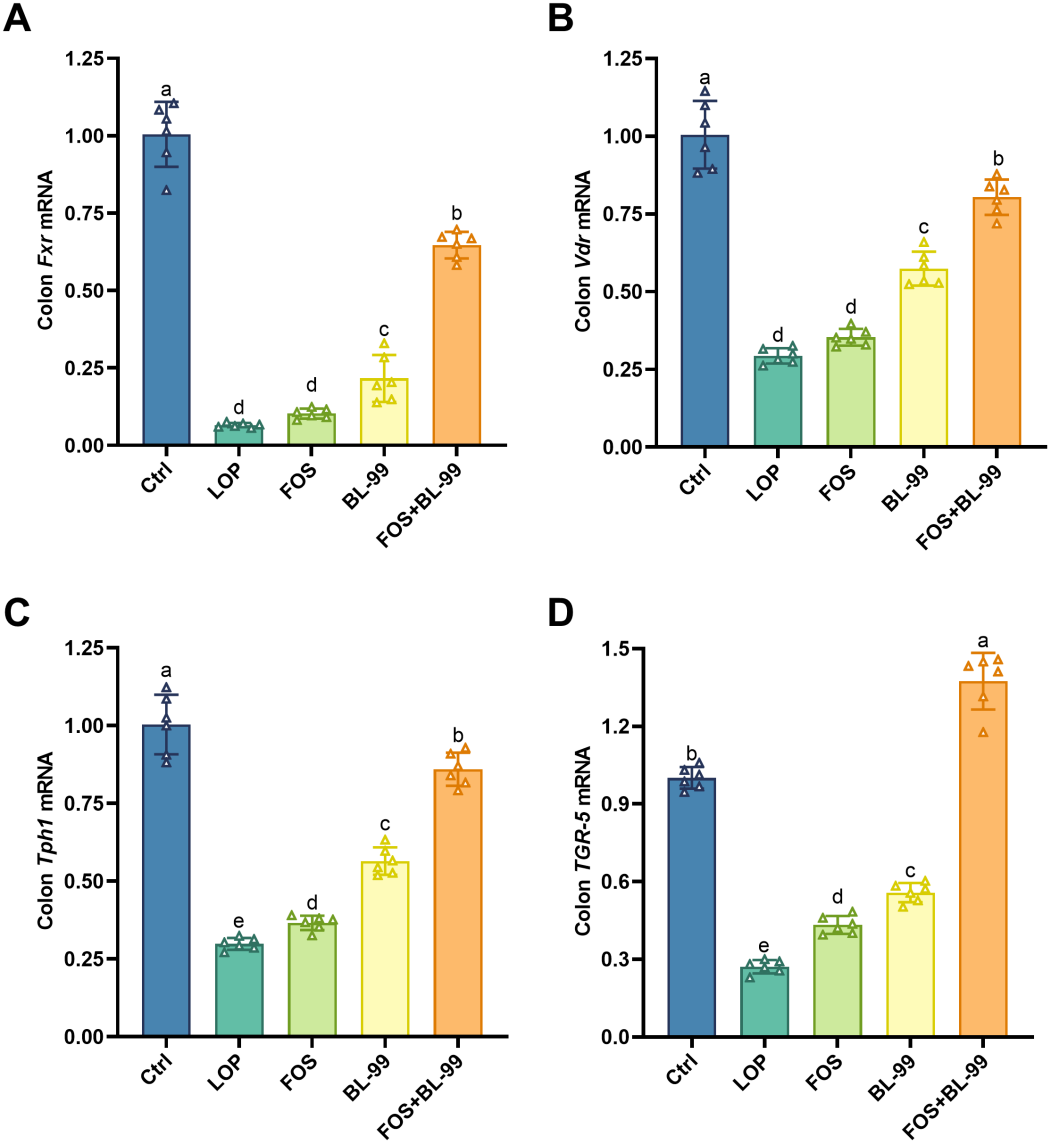
Effects of probiotic, prebiotic, and synbiotic on *Fxr* **(A)**, *Vdr* **(B)** *TGR5* **(C)**, and *Tph1* **(D)** mRNA expression levels. Values were expressed as M ± SD (n = 6). Results were compared by one-way ANOVA followed by LSD *post hoc* test. Different letters represent significant differences (*P* < 0.05).

The transcription levels of key genes in the 5-HT pathway within colonic tissues are crucial for maintaining intestinal health. As illustrated in **Figure 9**, the transcription levels of *Fxr*, *Vdr*, *TGR5*, and *Tph1* genes in the colonic tissues of mice in the LOP group were significantly reduced under constipated conditions compared to the Ctrl group. This reduction indicates a weakened defense against pathogens in the colon, thereby increasing the risk of colitis, a finding corroborated by the inflammation indicators shown in **Figure 4**. In contrast, the transcription levels of *TGR5* and *Tph1* genes were significantly elevated in both the BL-99 and FOS groups compared to the LOP group. Furthermore, the BL-99+FOS group exhibited a significant increase in the transcription levels of *Fxr*, *Vdr*, *TGR5*, and *Tph1* genes compared to the LOP, FOS, and BL-99 groups. This enhancement in gene transcription indicates a strengthened pathogen defense capability, reduced inflammation, and effective alleviation of constipation symptoms in mice.

## 4 Discussion

Constipation, a prevalent gastrointestinal condition, has affected a substantial portion of the global population (23). The systematic review reported that the incidence of constipation is higher in women than in men (1). Clinical studies have confirmed that synbiotics can significantly increase the number of bowel movements per week in constipation patients (4). In a previous *in vitro* fermentation study, the synergistic effects on modulating gut microbiota of people with constipation by the combination of BL-99 and FOS has been elucidated (20). Our investigation aimed to elucidate the alleviative potential of FOS, BL-99, and their synergistic combinations (synbiotics) on constipation *in vivo*. The experimental regimen, involving pretreatment with loperamide followed by oral administration of FOS and BL-99, either independently or in combination, yielded insightful findings. Notably, the intervention of FOS, BL-99, especially FOS+BL-99 demonstrated significant improvements in constipation-related parameters compared to the loperamide-induced constipation model, closely aligning with the physiological benchmarks observed in the non-constipated control group. These outcomes are highlighted by the marked enhancement in defecation metrics, including the first black stool defecation time and the gastrointestinal transit rate.

Recent studies have confirmed that intestinal neurotransmitters play a key role in the regulation of gastrointestinal motility, and their abnormal levels may lead to decreased colonic motility, which is an important pathogenesis of constipation (24). Intestinal neurotransmitters are mainly classified into excitatory and inhibitory types. Excitatory neurotransmitters include Ach, SP and 5-HT, which relieve constipation symptoms by stimulating smooth muscle contraction (25). Inhibitory neurotransmitters mainly include VIP and NOS, which mainly weaken gastrointestinal motility and cause difficulty in defecation (26). Liu G et al. found that in the constipation model, the expressions of NOS and VIP in the colon were significantly increased, while the expressions of Ach, SP and 5-HT were significantly decreased (27). According to the study of Zhang T et al (28), the intervention of *Bifidobacterium longum* S3 can significantly reduce VIP and NOS levels, and significantly increase 5-HT and Ach levels in constipation mice, which may play an important role in the pathogenesis of constipation. This study also found that BL-99+FOS significantly promoted the levels of excitatory neurotransmitter in serum of constipated mice, and decreased the inhibitory neurotransmitter, especially for the 5-HT level compared to the BL-99 and FOS groups. In conclusion, BL-99+FOS significantly promoted the serum 5-HT content, thus alleviating the symptoms of constipation.

The occurrence of constipation symptoms will change the intestinal permeability, lead to the breakdown of the intestinal immune system and the change of serum inflammatory factors (29). Some studies have reported higher concentrations of pro-inflammatory cytokines (such as IL-6 and TNF-α) and lower concentrations of anti-inflammatory cytokines (such as IL-10 and IL-4) in constipated individuals (30). Meanwhile, other studies confirmed that the intervention of probiotics (including *Lacticaseibacillus paracasei, Bifidobacterium lactis* et al.) (31, 32) or synbiotics (FOS and *Lactobacillus paracasei* LCP-31, *Lactobacillus rhamnosus* HN001, *Lactobacillus acidophilus* NCFM and *Bifidobacterium lactis* HN019) (33) can reduce the expression of IL-6 and TNF-α, and increase the expression of IL-10 and IL-4. In this study, gavage of loperamide led to changes in serum inflammatory factor levels. Compared with BL-99 or FOS alone, BL-99+FOS intervention significantly promoted serum IL-10 levels, indicating that BL-99+FOS may regulate intestinal peristalsis by improving the immune system.

In recent years, the correlation between gut microbiota and constipation has been increasingly recognized, and a large number of studies have confirmed that intestinal microbiota plays an important role in the occurrence of constipation (34). Mancabelli L et al (35) collected fecal samples from 79 healthy individuals and 147 constipation patients, and then, 16S-rRNA technology was used to determine fecal microbes; The results showed that the relative abundance of *Bacteroides, Roseburia* and *Coprococcus*_3 in constipated individuals stool were significantly lower compared with healthy individuals. *Lachnospiraceae_*NK4A136_group, a probiotic in the family *Lachnospiraceae*, can produce butyric acid during its growth, and its abundance level is negatively correlated with enteritis (36). *Bacteroides* are associated with opportunistic pathogens, especially peritoneal and systemic infectious pathogens (37). As a member of *Firmicutes*, *Blautia* shows promise in alleviating inflammatory and metabolic diseases because it has antibacterial activity against specific microorganisms and its abundance is positively correlated with acetic acid, propionic acid, and butyric acid content. In addition, some studies have shown that FOS can increase the abundance of *Blautia* (38). Meanwhile, the same study also reported that Latilactobacillus sakei Furu2019 and stachyose could relieve constipation trough the gut microbiota (39). The results of gut microbiota showed that the intervention of BL-99+FOS significantly increased the relative abundance of *Blautia*, and *Lachnospiraceae*_NK4A136_group in the caecum, while significantly decreased the relative abundance of *Bacteroides*. Therefore, BL-99+FOS intervention promotes intestinal motor function by regulating gut microbiota composition.

At the same time, a relationship between gut metabolites and constipation was also established (40). For example, SCFAs in the body is mainly the product of gut microbiota fermentation polysaccharide, resistant starch or oligosaccharides, etc. The increase of SCFAs in the intestine can regulate the intestinal pH value and regulate the intestinal immune system, thus promoting the colon peristalsis function (41, 42). In this study, BL-99+FOS promoted the formation of SCFAs, and the experimental results showed that the combination of BL-99 and FOS was beneficial to the development of the body in a healthy direction, and alleviated constipation by increasing the abundance of SCFAs. In addition, tryptophan metabolites can regulate colon peristaltic function in constipation patients by activating the release of neurotransmitters (43). In this study, it was found that the levels of tryptophan and its metabolites in the constipated mice cecum were significantly reduced, and the combined intervention of BL-99 and FOS could significantly up-regulate the levels of N-hydroxy-L-tryptophan, indole, and indole-3-aldehyde. In general, BL-99+FOS may improve constipation symptoms by regulating intestinal metabolites.

In light of these findings, our study further conducted an association analysis of gut microbiota with multiple indicators, confirming the utility of BL-99+FOS intervention in improving constipation mediated by multiple mechanisms, including regulation of gut microbiota composition and function, neurotransmitter synthesis, and inflammatory status. However, further investigations delineating the precise molecular underpinnings of these effects, as well as clinical trials corroborating these experimental insights, are imperative to fully harness the therapeutic potential of synbiotics in constipation management.

## 5 Conclusions

This study provides compelling evidence of the therapeutic potential of FOS, BL-99, and their synbiotic combination in treating constipation. The multi-faceted benefits observed underscore the synbiotic combination’s superiority, paving the way for novel dietary or therapeutic strategies aimed at managing constipation and improving gut health. Further investigations into the mechanisms underlying these effects will be instrumental in elucidating the full spectrum of benefits conferred by these interventions.

## Data Availability

The datasets presented in this study can be found in online repositories. The names of the repository/repositories and accession number(s) can be found below: https://www.ncbi.nlm.nih.gov/, PRJNA1141244.
